# Nitric Oxide in the Management of Respiratory Consequences in COVID-19: A Scoping Review of a Different Treatment Approach

**DOI:** 10.7759/cureus.23852

**Published:** 2022-04-05

**Authors:** Arunibha Ghosh, Betsy Joseph, Sukumaran Anil

**Affiliations:** 1 Neurosciences, S.N.Pradhan Centre for Neurosciences, University of Calcutta, Kolkata, IND; 2 Periodontics, Saveetha University, Chennai, IND; 3 Dentistry, Oral Health Institute, Hamad Medical Corporation, Doha, QAT; 4 Dentistry, College of Dental Medicine, Qatar University, Doha, QAT

**Keywords:** acute respiratory distress syndrome [ards], cytokine release storm, sars-cov-2, covid -19, anti-inflammation, cytokine storm syndrome, nitric oxide (no)

## Abstract

The severe acute respiratory distress syndrome coronavirus 2 (SARS-CoV-2) virus causing COVID-19 significantly affects the respiratory functions of infected individuals by massively disrupting the pulmonary oxygenation and activating the synthesis of proinflammatory cytokines, inducing severe oxidative stress, enhanced vascular permeability, and endothelial dysfunction which have rendered researchers and clinicians to depend on prophylactic treatment due to the unavailability of proper disease management approaches. Previous studies have indicated that nitric oxide (NO) application appears to be significant concerning the antiviral activities, antioxidant, and anti-inflammatory properties in relieving disease-related symptoms. To identify, explore, and map the literature on the role of nitric oxide in the management of respiratory consequences in COVID-19 through this scoping review, Preferred Reporting Items for Systematic Reviews and Meta-Analyses (PRISMA) guidelines were followed during the search to answer the focal question: "What are the potential uses of nitric oxide in the management of respiratory failure in COVID-19?" Administering exogenous NO in the form of inhaled gas or stimulating the system to produce NO appears to be a suitable option to manage COVID-19-induced pneumonia and respiratory illness. This treatment modality seems to attenuate respiratory distress among patients suffering from severe infections or patients with comorbidities. Exogenous NO at different doses effectively reduces systemic hyperinflammation and oxidative stress, improves arterial oxygenation, and restores pulmonary alveolar cellular integrity to prevent the lungs and other organs from further damage. This therapy could pave the way for better management of COVID-19 before the onset of disease-related complications.

## Introduction and background

The COVID-19 disease, caused by the novel severe acute respiratory distress syndrome coronavirus 2 (SARS-CoV-2), has devastated worldwide healthcare facilities. The increasing prevalence of COVID-19 disease involving all age groups has raised concerns over its evolution since December 2019, which has led to the alteration in classical signs and symptoms of the disease due to the emergence of different variants in the SARS-CoV-2 clan [1-3]. The SARS-CoV-2 infection is associated primarily with acute respiratory distress syndrome (ARDS), pneumonia, fever, cough, body ache, sore throat, and fatigue. One of the main features is the severe damage to the lung epithelium. Additionally, viral mRNA and proteins are identified in the cerebrospinal fluid (CSF) of infected individuals. This demonstrates the virus's potential for crossing the blood-brain barrier and infecting the respiratory centers of the brain stem, resulting in respiratory distress in COVID-19 patients [4-8]. Interestingly, the variety of clinical symptoms ranges from mild/moderate (81%) to severe (14%) to, in some cases, critical (5%). This suggests that several comorbidities like obesity, hypertension, diabetes, chronic obstructive pulmonary disease (COPD), cardiovascular disease, cerebrovascular disease, and autoimmune disease or immunosuppressed conditions contribute to the onset of severe COVID-19 disease [9]. SARS-CoV-2 attaches to the host cell membrane through type II transmembrane serine protease (TMPRSS2)-furin-elastase. Thus, it mediates the binding of TMPRSS2 to angiotensin-converting enzyme 2 (ACE2) receptors, which are localized on the host cell surface of target organs [10,11]. Studies identified that ACE2 receptors are highly expressed in type II alveoli epithelial cells, with 83% of cells positively staining for ACE2 receptors, which serve as the primary target for the viral attack [11,12]. Apart from that, the virus significantly impacts other organs with the development of myocarditis, gastrointestinal (GI) disturbances, renal ailments, and irregular blood pressure. The presence of ACE2 receptors on the epithelial and endothelial lining of the liver, heart, kidney, pancreas, GI tract, genital organs, thyroid, blood vessels, and so on is considered responsible for this [13,14].

COVID-19 patients can present with fever, cough, and shortness of breath which can lead to acute lung injury (ALI), ARDS, respiratory and multiorgan failure, sepsis or cardiac arrest, and eventually death [15]. Acute respiratory distress and hypoxemia arise due to severe exudation and hyaline membrane formation in alveolar spaces, bilateral diffuse alveolar damage with edema, pneumocyte desquamation, and massive pulmonary embolism [16,17]. Besides that, SARS-CoV-2 induces cellular senescence and apoptosis of epithelium and endothelium, further developing an array of hyperactivated and dysregulated host immune responses. These events can result in cytokine storm, which triggers acute lung injury by degrading cellular integrity and affecting the pulmonary microenvironment [18-21]. Hence, treatment should be targeted to inhibit viral replication, transcription or translation, and synthesis of new virions to alleviate symptoms of respiratory distress and protect the lungs epithelium from further damage. However, the absence of specific therapy necessitates the researchers and healthcare providers to depend on prophylactic measures to control viral replication and curb disease transmission across populations [15].

To achieve this, studies were conducted to understand the antiviral activity of ivermectin in the context of SARS-CoV-2 infection. This drug was previously identified with an antiparasitic effect and later established as an antiviral agent in limiting several RNA virus infections such as dengue virus (DENV) 1-4, West Nile virus, Venezuelan equine encephalitis virus (VEEV), and influenza virus [22]. Ivermectin demonstrated potential inhibition to SARS-CoV-2 replication in vitro and is associated with a lower mortality rate among hospitalized COVID-19 patients [22,23]. Thus, ivermectin appeared as an effective prophylactic treatment in minimizing the incidence of COVID-19 disease [24]. Moreover, a five-day course of ivermectin 12 mg treatment combined with doxycycline 200 mg on day one, followed by 100 mg every 12 h for the next four days, exhibited a significant decline in the duration of illness among mild-to-moderate COVID-19 cases in Bangladesh [25]. In addition to that, remdesivir, favipiravir, chloroquine, and hydroxychloroquine emerged as notable prophylactic treatments for lowering mortality and viral activity among patients [26,27]. According to a randomized, open-label, placebo-controlled trial, a five-day course of remdesivir intravenous injection proved to be more effective than a 10-day course of the same and placebo [28]. In studies, favipiravir similarly ameliorated symptoms of COVID-19 and reduced the duration of fever [27]. Besides that, more treatment measures with the help of bevacizumab and tocilizumab targeting the cytokine storm were attempted to prevent the release of interleukin 6 (IL-6) and other molecules, including vascular endothelial growth factor (VEGF) in the infected cells of the lung epithelium [27]. Randomized clinical trials suggested that tocilizumab, a monoclonal antibody against IL-6, effectively decreased the likelihood of undergoing mechanical ventilation in patients with COVID-19-induced pneumonia and hyperinflammation [29].

Both symptomatic and respiratory support are essential; however, management of COVID-19 requires further exploration of other approaches regarding attenuating oxidative stress to improve respiratory output among patients. Nitric oxide (NO) has been proved as an endothelium-derived vasodilator. It has been used to control vascular signaling, regulate blood flow, inflammation, and induce host defense. Its antioxidant activity enables the scavenging of reacting oxygen species (ROS) to maintain normal vascular function [15]. This scoping review aims to obtain insight into the potential use of nitric oxide in the management of respiratory failure in COVID-19 and how NO could emerge as a possible therapy in the future for this disease.

## Review

Methods

Search Strategy

This scoping review included observational, retrospective, prospective study and randomized controlled trials (RCTs) published in English since 1993 using the MeSH terms in combination with "COVID-19" or "SARS-CoV-2" or "respiratory distress" or "acute respiratory distress syndrome" or "pulmonary hypoxemia" or "respiratory illness" or COVID-19 pathogenesis" AND "COVID-19 treatment" or "nitric oxide" or "inhalation of nitric oxide" or "prophylactic treatment of COVID-19". PubMed/Medline, PMC, Google Scholar, and Scopus databases were searched to identify these articles which were selected based on their title, type of research paper, abstract, and findings, while conference papers and dissertations were not considered.

Aim of Scoping Review

The review focused on the use of nitric oxide and attempted to identify whether nitric oxide therapy can reduce the risk of respiratory illness, severe pneumonia or hospitalization, and the requirement of oxygen support among moderate-to-severe COVID-19 cases. To answer these questions, a review protocol was prepared following the Preferred Reporting Items for Systematic Reviews and Meta-Analyses (PRISMA) guidelines during the search and selection process. However, it was not registered in the International Prospective Register of Systematic Reviews (PROSPERO) because it was not developed as a systemic review. Eligibility criteria were decided before the commencement of the study. Study characteristics were identified based on the population of COVID-19 cases who were intervened with nitric oxide inhalation, and outcomes were estimated using significant clinical improvements.

Initial Selection

Titles and abstracts obtained from the electronic bibliographic database search were reviewed for potential eligibility by authors. Full texts were downloaded only for those studies which met the eligibility criteria. The guidelines given in the Cochrane Handbook for Systematic Reviews were used to give direction to the discussion. Study findings were extracted from articles by authors into an excel sheet. Disagreements between the authors were evaluated and resolved following discussion, and if necessary, evidence was subjected to further screening by other reviewers to avoid noncompliance with the guidelines.

Inclusion Criteria for the Target Population

The target population includes COVID-19 individuals (adults) with moderate-to-severe respiratory distress, ARDS, respiratory disease, or hypoxemia, and with or without a history of comorbidities. Additionally, those who required hospitalization during disease progression or did not recover despite effective preventative therapies with ivermectin, hydroxychloroquine, remdesivir, or tocilizumab were included. Research articles with evidence of the above-mentioned conditions and highlighting treatment effectivity of nitric oxide were selected for this review. Eligible articles demonstrating randomized controlled trials, nonrandomized controlled trials, cohort studies, case series, case studies, epidemiology articles, and sources reporting personal/expert opinions were included in actual cases.

Results

**Figure 1:**
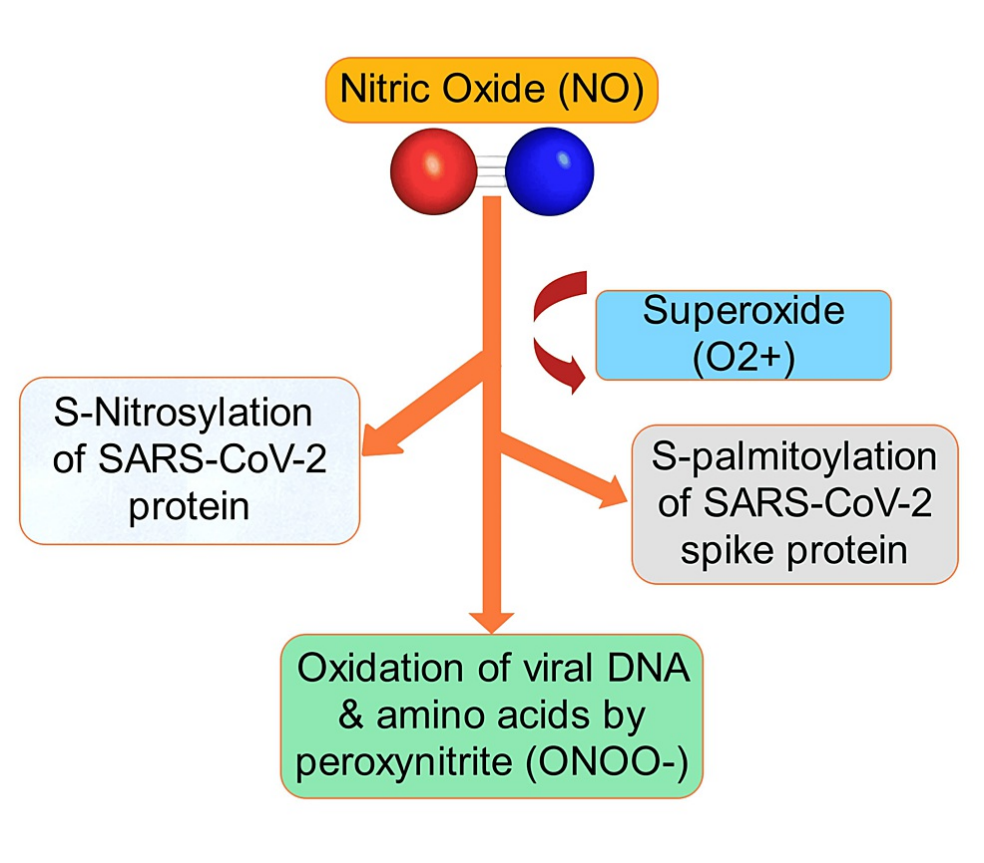
Mechanism of action depicting antiviral properties of inhaled nitric oxide SARS-CoV-2: severe acute respiratory distress syndrome coronavirus 2.

**Figure 2:**
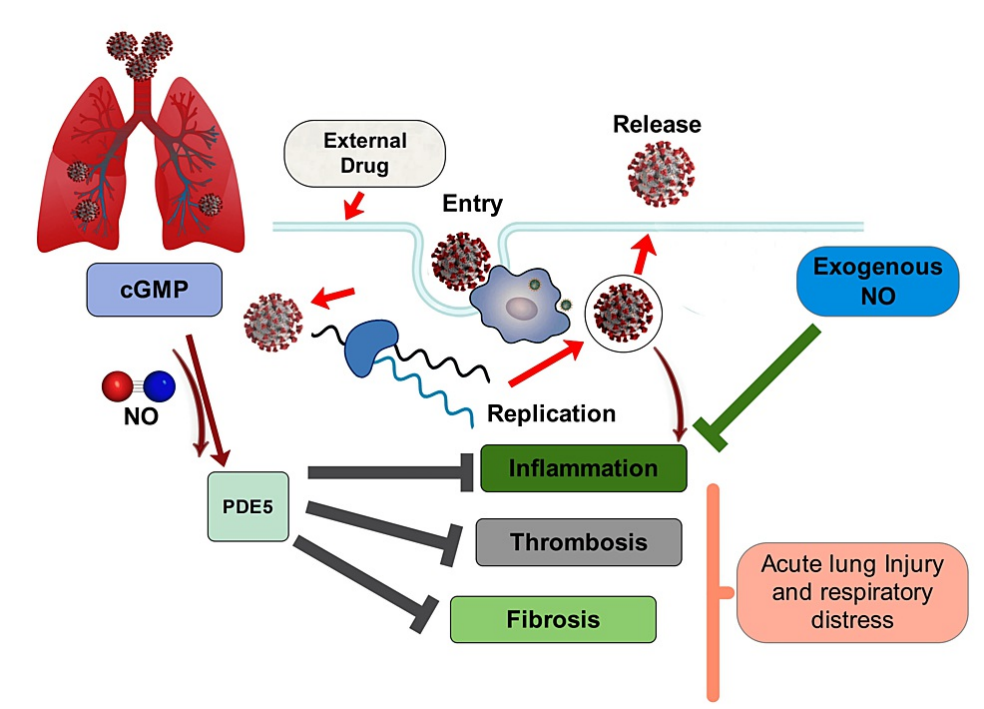
Exogenous nitric oxide and drug-induced NO production inhibits inflammation, fibrosis, and acute lung injury through cGMP-PDE5 mechanism cGMP: cyclic guanosine monophosphate, PDE5: phosphodiesterase type 5.

The analysis included a total of 43 studies investigating the use of nitric oxide therapy and other prophylactic measures performed on COVID-19 patients. The included studies covered prospective and retrospective studies, randomized controlled trials, and mechanistic approaches to unravel the background of effective nitric oxide therapy among patients. The literature covered a wide range of countries, like the USA, UK, Japan, Canada, Bangladesh, and China, with the earliest evidence being published in 1993 and the latest in 2021. A list of articles showing significant findings with respect to the use of inhaled NO and other prophylactic measures in the treatment of respiratory illness in COVID-19 is seen in Table 1. Figures 1, 2 depict the mechanism of action depicting antiviral properties of inhaled nitric oxide and inhibition of inflammation, fibrosis, and acute lung injury through cyclic guanosine monophosphate (cGMP)-phosphodiesterase type 5 (PDE5) mechanism.

**Table 1:** List of articles showing significant findings with respect to the use of inhaled NO and other prophylactic measures in the treatment of respiratory illness in COVID-19 SARS-CoV-2: severe acute respiratory distress syndrome coronavirus 2, ROS: reacting oxygen species, eNOS: endothelial nitric oxide synthase, COPD: chronic obstructive pulmonary disorder, IDO: indoleamine 2,3-dioxygenase, RCTs: randomized controlled trials, ppm: parts per million, α7nAChR: α7 nicotinic acetylcholine receptors, iNO: inhaled nitric oxide.

Record	Author, Year, Country	Title	Findings
1	Adusumilli et al. [15], 2020, USA	Harnessing nitric oxide for preventing, limiting and treating the severe pulmonary consequences of COVID-19	Antimicrobial and anti-inflammatory activities of NO are key to pulmonary vascular functions in the context of COVID-19
2	Xu et al. [17], 2020, China	Pathological findings of COVID-19 associated with acute respiratory distress syndrome	Pathologically, pulmonary edema, serous exudation, hyaline membrane, and alveolar damage cause ARDS in COVID-10
3	Wang et al. [21], 2020, China	Cytokine storm and leukocyte changes in mild versus severe SARS-CoV-2 infection: Review of 3939 COVID-19 patients in China and emerging pathogenesis and therapy concepts	Treatment strategies to address cytokine storm and other pathological changes in different stages of COVID-19
4	Caly et al. [22], 2020, Australia	The FDA-approved drug ivermectin inhibits the replication of SARS-CoV-2 in vitro	Antiviral effect of ivermectin presented in vitro along with antiparasitic effect
5	Rajter et al. [23], 2021, USA	Use of ivermectin Is associated with lower mortality in hospitalized patients with coronavirus disease 2019: the ivermectin in COVID nineteen study	Lower mortality achieved by using ivermectin in hospitalized COVID-19 patients
6	Hellwig and Maia. [24], 2021, USA	A COVID-19 prophylaxis? Lower incidence associated with prophylactic administration of ivermectin	Lower incidence of COVID-19 with the use of ivermectin
7	Ahmed et al. [25], 2021, Bangladesh, Indonesia	A five-day course of ivermectin for the treatment of COVID-19 may reduce the duration of illness	A five-day course of ivermectin reduced the duration of illness
8	Beigel et al. [26], 2020, USA	Remdesivir for the treatment of Covid-19: final report	In a double-blind, placebo-controlled randomized trial, remdesivir shortens the duration of recovery
9	Samudrala et al. [27], 2020, India	Virology, pathogenesis, diagnosis and in-line treatment of COVID-19	Prophylactic treatment improves the clinical outcomes of patients
10	Spinner et al. [28], 2020, USA	Effect of remdesivir vs standard care on clinical status at 11 days in patients with moderate COVID-19: a randomized clinical trial	A randomized, open-label controlled trial showed that a five-day course instead of a 10-day course of remdesivir improved the clinical outcome of patients
11	Salama et al. [29], 2021, USA	Tocilizumab in patients hospitalized with Covid-19 pneumonia	Reducing the likelihood of progression of composite outcome of mechanical ventilation
12	Rossaint et al. [30], 1993, USA, Germany	Inhaled nitric oxide for the adult respiratory distress syndrome	Inhaled NO decreases pulmonary artery pressure and increases pulmonary oxygenation
13	Izadi et al. [31], 2021, Iran, Italy, China	Ozone therapy for the treatment of COVID-19 pneumonia: a scoping review	Ozone therapy improves lung damage, reduces acute lung injury, ARDS
14	Akerstrom et al. [32], 2009, Sweden, Indonesia	Dual effect of nitric oxide on SARS-CoV replication: viral RNA production and palmitoylation of the S protein are affected	NO or its derivatives inhibit viral synthesis in the early stages of infection
15	Anderson and Reiter [33], 2020, UK, USA	COVID-19 pathophysiology: interactions of gut microbiome, melatonin, vitamin D, stress, kynurenine and the alpha 7 nicotinic receptor: treatment implications	Use of melatonin and α7nAChR agonist to mitigate pulmonary embolism in COVID-19
16	Belladonna and Orabona [34], 2020, Italy	Potential benefits of Tryptophan metabolism to the efficacy of Tocilizumab in COVID-19	Tocilizumab inhibits hyperinflammation by blocking IL-6 signaling
17	Turski et al. [35], 2020, Poland, Germany	AhR and IDO1 in pathogenesis of Covid-19 and the “Systemic AhR Activation Syndrome:” a translational review and therapeutic perspectives	Downregulation of AhR and IDO genes by dexamethasone, vitamin D, and vitamin E reduces the risk of SARS-CoV-2 infection
18	Ritz et al. [36], 2021, USA	Boosting nitric oxide in stress and respiratory infection: potential relevance for asthma and COVID-19	NO provides benefits for patients suffering from asthma, lung infection, and SARS-CoV-2 infection
19	Tejero et al. [37], 2019, USA	Sources of vascular nitric oxide and reactive oxygen species and their regulation	NO reduces ROS generation
20	Akerstrom et al. [38], 2005, Sweden	Nitric oxide inhibits the replication cycle of severe acute respiratory syndrome coronavirus	NO donor, S-nitroso-N-acetylpenicillamine, inhibited the replication cycle of SARS-CoV in a concentration-dependent manner
21	Pieretti et al. [39], 2021, Brazil, Chile	Nitric oxide (NO) and nanoparticles: potential small tools for the war against COVID-19 and other human coronavirus infections	Delivery of localized NO by nanomaterials to improve the immunological system of COVID-19 patients
22	Akaberi et al. [40], 2020, Sweden	Mitigation of the replication of SARS-CoV-2 by nitric oxide in vitro	NO donor, S-nitroso-N-acetylpenicillamine, dose dependently inhibited SARS-CoV-2 replication in vitro
23	Csoma et al. [41], 2019, Hungary	Dysregulation of the endothelial nitric oxide pathway is associated with airway inflammation in COPD	Systemic steroid treatment reverts impairment in eNOS function in COPD and other lung diseases
24	Zhou et al. [42], 2020, France	Challenging development of storable particles for oral delivery of a physiological nitric oxide donor	NO donors and especially S-nitrosothiols such as S-nitrosoglutathione (GSNO) facilitate oral delivery of NO
25	Thomas et al. [43], 1994, Australia	Nitric oxide inhibits indoleamine 2,3-dioxygenase activity in interferon-gamma primed mononuclear phagocytes	NO inducing inhibition to IDO activity in phagocytes beneficiary in lung inflammation
26	Lei et al. [44], 2020, China, USA	Protocol of a randomized controlled trial testing inhaled nitric oxide in mechanically ventilated patients with severe acute respiratory syndrome in COVID-19 (SARS-CoV-2)	Protocol developed to treat COVID-19 patients with NO in RCTs
27	Gianni et al. [45], 2020, USA	Nitric oxide gas inhalation to prevent COVID-2019 in healthcare providers	Inhalation of NO to prevent COVID-19 among healthcare workers
28	Ferrari et al. [46], 2020, Italy	Inhaled nitric oxide in mechanically ventilated patients with COVID-19	iNO relieves hypoxemia in mechanically ventilated COVID-19 patients
29	Tavazzi et al. [47], 2020, Italy	Inhaled nitric oxide in patients admitted to intensive care unit with COVID-19 pneumonia	iNO induced improvement in oxygenation and cardiac output
30	Parikh et al. [48], 2020, USA	Inhaled nitric oxide treatment in spontaneously breathing COVID-19 patients	iNO reduced the likelihood of mechanical ventilation
31	Safaee Fakhr et al. [49], 2020, USA	High concentrations of nitric oxide inhalation therapy in pregnant patients with severe coronavirus disease 2019	NO at 160-200 ppm beneficiary for pregnant

Significant findings

Manifestation of COVID-19 is mediated through the hyperactivation of the host immune system, macrophage infiltration, and recruitment of dysregulated T cells against the viral infestation. Besides, stimulation of excessive ROS generation through neutrophil recruitment and an abnormal NO/ROS ratio expedites the disease with the presentation of severe pneumonia, cytokine storm, respiratory distress, and hypoxemia, as demonstrated by researchers [15,27,30]. Detailed investigations involving the application of NO reveal that exogenous NO inhibits replication, transcription, and protein synthesis of the SARS-CoV-2 along with other viruses, depletes the cytokine storm, and acts as a vasodilator with anti-inflammatory properties by downregulating the activity of angiotensin-2 (AT-2) and scavenging the reactive metabolites from infected cells [31,32,50-52]. Moreover, researchers observed that induction of NO protects RBCs and hemoglobin from undergoing hemolysis and oxidative damage to reduce the onset of anemia among patients [14,15,53]. Administration of drugs to facilitate NO-mediated inhibition of phosphodiesterase type 5 (PDE5) pathway and endogenous synthesis of NO inactivates the indoleamine 2,3-dioxygenase (IDO) enzymatic activity causing further decline in hyperinflammatory response and improving pulmonary oxygenation and endothelial functionality to mitigate the symptoms of COVID-19 pneumonia [34-36].

Application of NO in COVID-19 Therapy

Management of COVID-19 was involved with different strategies of nitric oxide administration under different conditions. Researchers suggested the application of high doses of gaseous NO, up to 180 parts per million (ppm), during short periods of time (20-30 min), and low doses of gaseous NO, up to 80 ppm, during 48-h treatment for severe COVID-19 patients [44]. Intriguingly, other protocols recommended using inhaled NO for healthcare providers to prevent them from getting infected with the SARS-CoV-2 during their responsibilities. However, individuals susceptible to increased contact with COVID-19 patients and subsequent infection were administered a maximum of 160 ppm of inhaled NO for a short period of time during 14 days, which resulted in a decline in contamination from 15% to 5% [43]. According to reports, inhaled NO trials involving severe COVID-19 patients with invasive mechanical ventilation revealed that doses of 20 ppm for 30 min improved arterial oxygenation among patients. However, exceptions were observed in breathing patients with nonsevere conditions and those suffering from severe hypoxemia, which highlighted the significance of NO in the treatment of COVID-19 cases [46]. Tavazzi et al. [47] demonstrated complementary and encouraging results with the application of inhaled NO at a concentration of 25 ppm among COVID-19 patients having right ventricular dysfunction, which, contradictorily, showed no improvement in oxygenation among patients encountering refractory hypoxemia. Interestingly, a different study investigating the impact of inhaled NO on 39 COVID-19 patients with or without preexisting health conditions revealed that a dosage of 30 ppm of NO for two days ameliorated the oxygen saturation (SpO_2_)/fraction of inspired oxygen (FiO_2_) ratio in 53.9% of patients without requiring mechanical ventilation [48]. In addition to that, a higher dose of NO at 200 ppm for 30 min of duration diminished respiratory failure demonstrating an improvement in cardiopulmonary function among six pregnant COVID-19 patients who underwent a total of 39 treatments for COVID-19 and subsequently were discharged from the hospital following treatment with higher doses of inhaled NO [49]. This indicated the significant antiviral activity of NO apart from the prevention of hypoxic respiratory failure [48].

Discussion

Pathogenesis of COVID-19

Pathophysiology of COVID-19 is associated with multiple pathways pertaining to higher infectivity of the SARS-CoV-2, which influences host immune defense in favor of its survival. Researchers suggested that SARS-CoV-2 has evolved with increased pathogenicity compared to its previous counterparts, SARS-CoV and Middle East respiratory syndrome coronavirus (MERS-CoV), due to several mutations in the receptor-binding domain (RBD) of the spike (S) protein. As a result, furin-mediated cleavage of the polybasic site, RRAR, at the junction of S1/S2 of SARS-CoV-2 generates two noncovalently attached spike proteins, S1 and S2, which increase pathogenicity, infectivity, and tropism of the virus [48]. As mentioned earlier, COVID-19 manifests with pathological features of acute lung injury, bilateral diffuse alveolar damage with edema, hyaline membrane formation within alveolar spaces, cytokine storm, massive pulmonary embolism, and apoptosis epithelium and endothelium, leading to the appearance of respiratory distress and pneumonia. Subsequent vascular permeability, dysregulated T-cell response, and aggravated immune system result in hyperinflammatory response and induce an abnormal NO/ROS ratio. In addition to that, SARS-CoV-2 attaches to the dendritic cells of the host through its antigen-presenting ability and activates M1 macrophage to induce excessive ROS generation through recruitment of neutrophils and enhancing the production of peroxynitrite along with NO [15,27]. These cascades of reactions enable the synthesis and release of numerous proinflammatory cytokines, including interferon (IFN)-α, IFN-γ, interleukin (IL)-1β, IL-6, IL-12, IL-18, IL-33, tumor necrosis factor (TNF)-α, and transforming growth factor (TGF)-β, and chemokines, like chemokine ligand (CCL) 2, CCL3, CCL5, CXC chemokine ligand (CXCL) 8, CXCL9, and CXCL10, in the form of "cytokine storm" further neutralizing the virus and contributing to the collateral damage of endothelial dysfunction, permeable vessels, and lipid membrane peroxidation [15,27,37]. Incidentally, SARS-CoV-2 has been reported to cause hemolysis resulting in the onset of anemia and increased coagulation among patients by scavenging endothelial NO, proinflammatory heme, and activating platelets which eventually trigger an enhanced ROS generation and exacerbate immune response [15,53-55]. Reduced blood flow generates hypoxia giving rise to impaired organ function due to the formation of clots within blood vessels. However, the synthesis of NO converts M1 macrophages to the population of M2 macrophages to prevent organs from further damage by the proinflammatory response. It later initiates a repair process to aid in the clearance of cellular debris.

SARS-CoV-2 entry into host cells is mediated by binding to ACE2 receptors through enzymatic cleavage of the spike protein involving furin-TMPRSS2-elastase, which is located on the host cell surface [10,11,56]. Apart from that, ACE2, an enzyme in the renin-angiotensin system (RAS), serves the function of vasodilation and anti-inflammation through the conversion of angiotensin-2 (AT-2) to angiotensin 1-7, which is responsible for increased production of NO [50]. AT-2 has been identified as a potent vasoconstrictor to induce oxidative stress by activating nicotinamide adenine dinucleotide phosphate (NADPH) oxidase, which contributes to the evolution of superoxide radicals [31,51,52]. Furthermore, in the presence of oxidative stress, the antioxidant and anti-inflammatory properties of ACE2 contribute to the protection and maintenance of cellular integrity through inhibition of nuclear factor κβ (NF-κβ) pathway [50,57]. However, the binding of SARS-CoV-2 to ACE2 facilitated by viral spike protein causes virus-receptor complex internalization and subsequent ACE2 downregulation in infected cells [58]. Hence, successful viral invasion and ACE2 downregulation trigger the NF-κβ pathway, leading to mitogen-activated protein kinase p38 (p38/MAPK) activation, a proliferated level of cytokine release, and enhanced synthesis of AT-2 [57,59,60]. Increased production of AT-2 in the absence of inhibition by ACE2 potentially elevates pulmonary vascular permeability resulting in the formation of edema, hyaline membrane around edema, and development of acute lung injury [61]. Apart from that, researchers demonstrated that the activated p38 in the MAPK pathway is associated with cell growth arrest influencing cellular senescence by stimulating the expression of senescence inducers [62], the onset of which was further delayed by inhibition of SB203580.

Production of NO Associated With Viral Inhibition

The synthesis of nitric oxide (NO) originating from arginine in mammalian cells is mediated by three enzymes, neuronal nitric oxide synthase (nNOS), endothelial nitric oxide synthase (eNOS), and inducible nitric oxide synthase (iNOS). Elevated NO level induced by SARS-CoV-2 invasion functions as inhibitory to viral replication and viral protein accumulation; in addition, interference to S protein fusion, bronchodilation, and virus release from infected cells is facilitated by NO [58-60]. According to previous studies involving the NO donor, S-nitroso-N-acetylpenicillamine (SNAP), NO was able to inhibit the replication cycle of SARS-CoV, its protein, and RNA synthesis in a concentration-dependent mechanism [32]. However, the replication cycle inhibition is not mediated by the intermediate peroxynitrite, rather involves two mechanistic ways: interference in the fusion of S protein and its receptor due to reduced palmitoylation of the S protein, and a reduction in RNA production, probably due to an effect on cysteine proteases [38,40]. Evidently, the presence of NO in blood vessels and endothelial cells enables nitrosation of reactive thiols on the surface of RBCs and on the beta chain of the hemoglobin tetramer to prevent these cells from undergoing further hemolysis and oxidative damage, which are indicative of its potentiality to control virus-mediated RBC destruction and subsequent anemia [15,53,55]. However, during viral infection, a depleted NO accelerates systemic inflammation and impaired scavenging of ROS by disrupting oxygenation in blood and other organs, leading to hypoxia. Researchers have demonstrated that supplementation of NO diminishes cytokine storm and restores the functionality of blood vessels by scavenging reactive metabolites from tissues and hence mitigates the propagation of disease manifestation [30]. Incidentally, endogenously synthesized NO gradually decreases with increasing age and with the presence of comorbidities like obesity, type 2 diabetes, vascular inflammation, chronic obstructive pulmonary disorder (COPD), or autoimmune diseases, which require restoration of NO by external supplementation to prevent COVID-19 patients from succumbing to death due to ARDS and respiratory failure [30,41,63-65].

Potentiality of NO in Reducing Cytokine Storm, Inflammation, and Respiratory Distress

Circulating NO levels can be elevated by providing exogenous NO in inhaled gas, dietary supplementation, and administration of direct or indirect donors. Studies have indicated that inhaled gaseous NO's anti-inflammatory and vasodilator properties result in effective attenuation of acute respiratory distress among COVID-19 patients [41,42]. However, complex physiological pathways involved with NO production, its regulation, administration of external NO and its storage, high costs, and production of toxic by-products during NO delivery, such as NO_2_ and O_3_, have restricted its use by exacerbating severe hypoxic conditions and pulmonary hypertension. Interestingly, inflammatory mediators and the activity of important heme-containing enzymes, such as indoleamine 2,3-dioxygenase (IDO), are implicated toward the onset of inflammatory response and "cytokine storm" which are inhibited by the administration of NO [34]. Due to their strong affinity for IDO, NO and NO donors are thought to be cytosolic immune-modulatory enzymes containing heme iron that are inactivated by IDO [43]. Incidentally, monocyte activation drives the expression of IDO and inducible nitric oxide synthase (iNOS) to further elicit the iNOS-mediated release of NO [38]. Previously, the synthesis of NO has been shown to inhibit the SARS-CoV replication cycle through the regulation of inflammatory response by an antiviral mechanism of interferon (IFN)-γ, tumor necrosis factor (TNF)-α, and interleukins 6 (IL-6) and 1β (IL-1β) [35]. Activation of cytokines stimulates IDO enzymatic activity to perform tryptophan catabolism which controls the propagation of pulmonary hyperinflammation, hypertension, and pneumonia among COVID-19 patients with its immunoregulatory and antimicrobial activities [34,66,67]. Hence, these series of events attempt to accelerate the synthesis of NO to hinder IDO enzymatic activity and excessive release of cytokines.

Several medications have been addressed in this context, with some drugs increasing the synthesis of NO through cGMP-mediated inhibition by phosphodiesterase type 5 (PDE5) [68,69]. Evidently, soluble organic nitrates have originated as indirect NO donors being used in the treatment of cardiovascular diseases showing a significant number of deleterious effects. Studies have recommended the use of leafy vegetables comprising a high amount of inorganic nitrate and nitrites, which ameliorate age-related endothelial dysfunction with its potential anti-inflammatory activities and exert vascular and immunological benefits [70]. The involvement of NO-cyclic GMP-phosphodiesterase type 5 (PDE5) pathway was demonstrated previously by a group of researchers where PDE5 inhibition provided an anti-inflammatory response by influencing activated T cells to reduce cytokine (IL-6) release and subsequently diminished pulmonary fibrosis with improvement in oxygenation and stimulation of vascular repair. In addition to offering protection against metabolic and cardiovascular diseases, these PDE5 inhibitors were predicted to be associated with the management of COVID-19 by impeding angiotensin-II-driven downregulation of AT-2 receptors, which induced monocyte switching to alleviate cytokine storm, interstitial infiltration, and blood vessel damage [34]. Consequently, this would inhibit the formation of mesenchymal cells from the endothelial and smooth muscle cells of the pulmonary artery to prevent thrombotic complications and necrosis of hemorrhagic alveoli [34]. Interestingly, a different study exhibited that the continuous nitric oxide release from S-nitroso-N-acetyl-D, L-penicillamine led to a decline in lung macrophage recruitment and their infiltration inside alveolar spaces reducing the synthesis of TNF-α and IL-1β in a dose-dependent manner [32]. Hence, NO administration was implicated toward the depletion in cytokine storm associated with the recovery from acute lung injury following virus-induced inflammation and respiratory distress.

## Conclusions

This review elaborated on the potential application of nitric oxide therapy in modifying the immune response of COVID-19 patients through the prevention of hyperinflammatory response and cytokine storm by inducing host immune defense against viral invasion and reducing cellular oxidative stress, which would mitigate the propagation of COVID-19 disease. Exogenous NO was shown to effectively reduce systemic hyperinflammation, oxidative stress through inhibition of IDO enzymatic activity, downregulation of AT-2 and its receptors, and inactivation of cGMP-mediated PDE5 pathway among severe COVID-19 patients. In summary, the application of exogenous nitric oxide could emerge as a prospective therapeutic approach in the prevention of respiratory consequences in COVID-19 and further worsening of disease conditions.
